# Downregulated Expression of the IL7R and BACH2 Genes Is Associated with Immune Memory Loss in Adults Vaccinated Against HBV at Birth

**DOI:** 10.3390/cimb48010047

**Published:** 2025-12-29

**Authors:** Ge Zhong, Zhi-Hua Jiang, Mei-Lin Huang, Xue-Yan Wang, Li-Ping Hu, Qin-Yan Chen, Lu-Juan Zhang, Yu-Bi Huang, Xue Hu, Rui-Min Li, Wei-Wen Zhou, Ying Huang, Sha Li, Tim J. Harrison, Zhong-Liao Fang

**Affiliations:** 1 Guangxi Key Laboratory for the Prevention and Control of Viral Hepatitis, Guangxi Zhuang Autonomous Region Center for Disease Prevention and Control, 18 Jin Zhou Road, Nanning 530028, China; 2 School of Preclinical Medicine, Guangxi Medical University, Nanning 530021, China; 3 Division of Medicine, University College London Medical School, London WC1E 6BT, UK

**Keywords:** hepatitis B virus, HepB vaccination, immune memory, transcriptome, single-cell RNA sequencing

## Abstract

Immunization is the most effective way to prevent transmission of the hepatitis B virus. However, about one-quarter of hepatitis B vaccinees (HepB vaccinees) aged around 18 years have lost their immune memory. What is responsible for the loss? Five subjects who became asymptomatic HBsAg carriers after anti-HBs seroconversion and ten controls who were negative for both HBsAg and anti-HBs were recruited from individuals born in 1987 and vaccinated at birth. scRNA-seq was performed on peripheral blood mononuclear cells, including library preparation, sequencing, quality control and filtering, normalization, dimensionality reduction, clustering, cell type annotation, differential expression analysis and trajectory analysis. Twelve cell types and nine subpopulations of T cells were identified. No significant differences in the proportions of cell types and subpopulations were found between cases and controls. The expression levels of immune memory-related genes, IL7R in total T cells and BACH2 in naive CD4+ T cells and naive CD8+ T cells, were significantly downregulated in the cases (*p* = 2.2 × 10^−308^, 3.31 × 10^−27^ and 9.41 × 10^−100^, respectively). IL7R is expressed throughout cellular development, while BACH2 is expressed only in the early stage of cellular development. Downregulation of the IL7R and BACH2 in T cells is associated with immune memory loss, identifying them as candidate genes for future functional studies to explore their potential role in the loss of immune memory. This could inform adjuvant design if a causal mechanism is firmly established.

## 1. Introduction

Chronic hepatitis B virus (HBV) infection remains a global public health problem. HBV infection may result in a spectrum of outcomes, including acute self-limited infection, acute and chronic hepatitis, liver cirrhosis and hepatocellular carcinoma (HCC) [1]. An estimated 250 million individuals are persistently infected with HBV, causing an estimated 887,000 deaths annually, mostly due to the long-term sequelae liver cirrhosis and HCC [2].

Immunization is the most effective means of preventing transmission of HBV. Seroprotection after vaccination, defined as neutralizing hepatitis B surface antibody (anti-HBs) ≥10 mIU/mL, is achieved in over 95% of vaccinees [3]. Neonatal HBV vaccination provides effective protection for at least 37 years [4]. However, over time, the titers of anti-HBs in vaccinated individuals decline, becoming undetectable in some. Furthermore, we and others have reported that some vaccinees become persistently infected, although they were positive for anti-HBs after vaccination against HBV at birth [5,6]. Others have found that the prevalence of HBsAg among the population immunized with hepatitis B (HepB) vaccine at birth is increasing with age [5,7,8,9], suggesting that they have lost immune memory.

In general, among the subjects who were vaccinated against HBV at birth and then tested positive for anti-HBs (≥10 mIU/mL), those who were found to be negative (<10 mIU/mL) or positive (≥10 mIU/mL) for anti-HBs after a booster dose of vaccine were considered to have immune memory loss and persisting immunity, respectively [10]. It has been reported that long-term immunity is provided by memory B cells and T cells in the blood and lymph nodes, as well as by long-lived plasma cells and memory T cells in the bone marrow [11]. However, reasons for the waning of immunity remain poorly understood.

Single-cell RNA sequencing (scRNA-seq) offers novel approaches for resolving the complexity and heterogeneity of immune cells, enabling the identification of novel cell subsets and the exploration of underlying cell lineage relationships [12]. In this study, based on the LongAn cohort [9], LongAn county, Guangxi, China, the transcriptional profiles of peripheral blood mononuclear cells (PBMCs) from vaccine recipients who became asymptomic HBsAg carriers and others who remained uninfected after becoming anti-HBs negative, were analyzed using scRNA-seq, with the purpose of searching for the factors associated with immune memory loss.

## 2. Materials and Methods

### 2.1. Study Subjects

LongAn county hosts one of the five clinical trials of hepatitis B immunization in China. In order to evaluate the efficacy of vaccination, a serial study has been carried out in LongAn since the early 1990s. The candidate study subjects were those who were born between 1987 and 1993 and vaccinated at birth with three doses of plasma-derived HepB vaccine and who have attended for evaluation and provided serum samples at least once in the last ten years. Individuals who became asymptomatic, chronic HBsAg carriers were selected as cases, while others who were negative for both HBsAg and anti-HBs were selected as controls. Doctors from local town hospitals and village clinics notified all candidate study subjects. Those who were willing to attend our visit became study subjects, completed a one-page questionnaire at that visit, and provided a 15 mL sample of blood by venipuncture for testing for serological markers of HBV, HBV DNA measurement and for scRNA-seq. The questionnaire included demographic information, sex, birth date, ethnicity, place of birth, and immunization history, as well as the history of diseases such as autoimmune liver disease, metabolic liver disease and generalized metabolic disorders, and blood transfusion.

Informed consent in writing was obtained from each individual. The study protocol conforms to the ethical guidelines of the 1975 Helsinki Declaration and has been approved by the Guangxi Institutional Review Board (Approval code: GXCDCIRB2024-0043; Approval date: 22 August 2024).

### 2.2. Qualitative Assays of HBV Serological Markers

Sera were tested for HBV serological markers, HBsAg, anti-HBs, HBeAg, anti-HBe and anti-HBc, using enzyme immunoassays (WANTAI BioPharm, Beijing, China). Quality control for the measurements was performed in accordance with the protocols provided by the manufacturer.

### 2.3. Collection of Peripheral Blood Mononuclear Cells (PBMCs)

Two mL of the peripheral blood samples were collected into EDTA anticoagulation tubes and processed immediately. PBMCs were separated by density gradient centrifugation, using Ficoll-Paque PREMIUM 1.084 g/L sterile solution (GE Healthcare, Uppsala, Sweden). Then, 90% FBS + 10% DMSO was added to the cryopreservation tube. The cells were stored at −80 °C for later analysis.

### 2.4. Construction of Libraries and Single-Cell RNA Sequencing

The mRNA for single-cell libraries was processed according to the DNBelab C Series Single Cell RNA Library Preparation Kit (MGI, Shenzhen, China). In brief, single-cell suspensions were subjected to a series of processes, including cell (nucleus) morphology inspection, droplets formation, homogenization, reverse transcription, cDNA double-strand synthesis, cDNA and oligo product amplification, intermediate product quality inspection, oligo library construction, cDNA product fragmentation, end repair, and addition of “A”, cDNA product adapter ligation, cDNA product PCR amplification, library quality control, and cDNA and oligo product circularization. PCR products were denatured into single strands, followed by circularization and reactions to obtain single-stranded circular products. Linear DNA molecules that had not been circularized were digested. Single-stranded circular DNA molecules underwent rolling circle replication, forming DNA nanoballs (DNBs) containing multiple copies. The obtained DNBs were applied to a high-density DNA nanochip, inserted into meshed pores on the chip, and sequenced using combinatorial probe–anchor synthesis (cPAS) technology on the DNBSEQ-G400 platform.

### 2.5. Quality Control and Determination of Major Cell Types in Single-Cell Data

The raw sequencing data were analyzed to generate a gene expression matrix using DNBC4tools (version v2.1.1). Subsequent downstream analysis was conducted using the R package Seurat (version 5.2.1) [13]. The quality control of cells was carried out, based on the number of genes detected and the proportion of mitochondrial reads according to the following criteria: (1) Cells with fewer than 200 genes identified or cells where the counts for the top gene constituted >90% of the total UMI count were removed; and (2) Cells were sorted in descending order based on the proportion of mitochondrial reads, and the top 15% were filtered out. Doublets were identified and removed using DoubletDetection. Cell cycle analysis was performed using the CellCycleScoring function in Seurat. The gene expression dataset was normalized, followed by PCA analysis using 2000 variable genes (n = 15). Subsequently, dimensionality reduction and clustering were conducted using the FindClusters function in the Seurat package (resolution 0.5). For each cluster, marker genes were identified using the FindAllMarkers function in the Seurat package. Differential gene expression analysis was performed between each cluster and all other cells to obtain marker genes. Differential gene expression was determined using the FindMarkers function (logfc > 0.25, minPct > 0.25, Padj ≤ 0.05, test.use = wilcox).

### 2.6. Functional Analysis of Marker Genes and Differential Genes

The clusterProfiler package (version 4.14.6) in R (version 4.4.3) was used to perform gene ontology (GO) enrichment analysis and Kyoto Encyclopedia of Genes and Genomes (KEGG) pathway analysis of differentially expressed genes. The analyses were based on the org.Hs.eg.db database (version 3.20.0) for human gene annotations. For KEGG enrichment analysis, the human KEGG database (hsa) was utilized. GO terms or pathways with FDR < 0.05 were considered significantly enriched.

### 2.7. Developmental Trajectory Analysis

Developmental trajectory analysis was performed using the Monocle 2 package (Version 2.34.0) [14]. Monocle 2 utilizes reverse graph embedding to describe multiple fate decisions in a completely unsupervised manner.

### 2.8. Statistical Analyses

R (version 4.4.3) and GraphPad Prism (version 8, La Jolla, CA, USA) were used for statistical analyses. Continuous variables are expressed as the median. Categorical data were evaluated by χ^2^ or Fisher’s exact tests, depending on the absolute numbers included in the analysis, and quantitative data were analyzed by the independent sample t-test or Mann–Whitney U test. The results are considered statistically significant if the adjusted *p* value is <0.05.

## 3. Results

### 3.1. General Characteristics of the Study Subjects

The 15 subjects in the study included 5 who became asymptomatic, chronic HBsAg carriers and 10 who were negative for both HBsAg and anti-HBs. The average age was 34.9 ± 1.8 (Mean ± SD) years. The average ages of the case and control groups were 35.2 ± 1.8 and 34.2 ± 1.8, respectively (Table 1).

### 3.2. Single-Cell Transcriptome Map of PBMCs from Adults Vaccinated Against HBV at Birth

**Figure 1:**
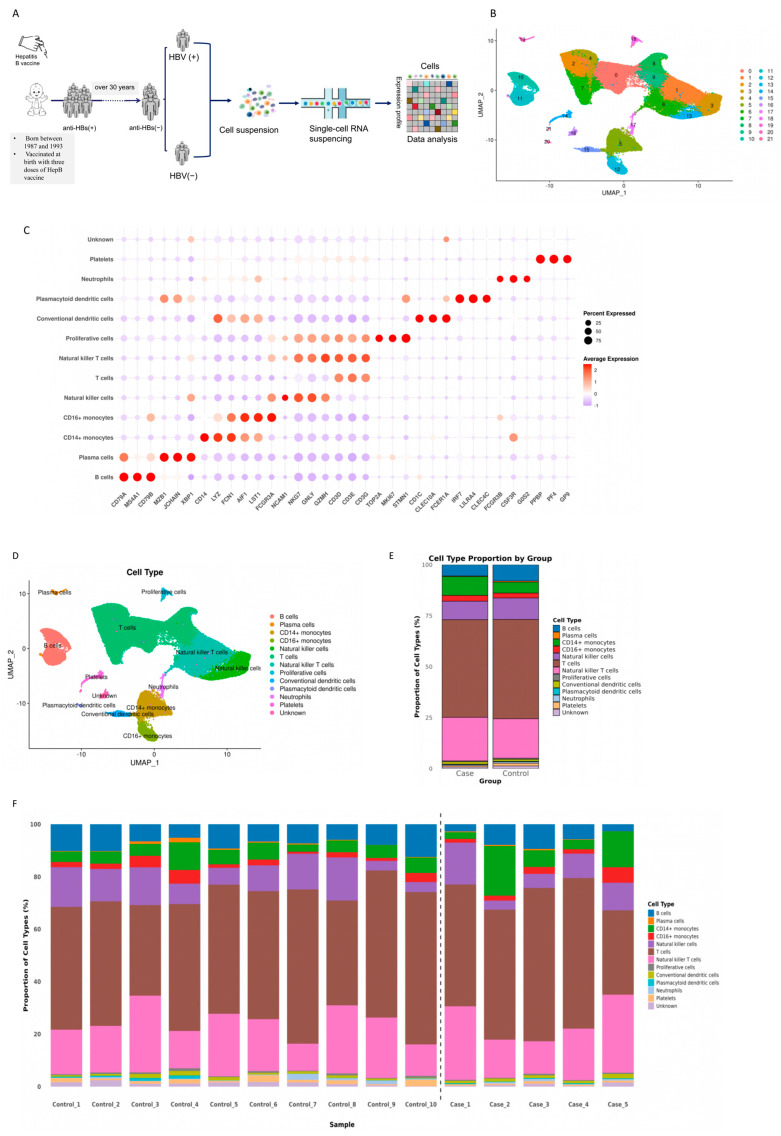
Single-cell transcriptome map of PBMCs from adults vaccinated against HBV at birth: (**A**) schematic workflow of experimental design and data analysis in this study; (**B**) UMAP plot of the 120,876 single cells from 15 individuals, revealing 22 distinct cell clusters. All 15 samples were merged and analyzed using Harmony for integration after normalization, variable feature selection, and scaling with the Seurat package. The clusters were identified through unsupervised clustering and visualized using UMAP; (**C**) dot plots showing the expression of key marker genes (x-axis) across all major cell types (y-axis) in the integrated dataset from all subjects. The depth of the color from light to dark and the size of the dots represent the average expression from low to high and the percentage of cells expressing the gene; (**D**) UMAP plot visualizing the integrated distribution of the 12 identified peripheral blood cell lineages across all samples; (**E**) the bar chart displays the proportion of different cell types in case group and control group; and (**F**) proportions of each cell type in each sample, colored according to the cell type.

To explore the cellular the heterogeneity of immune memory-related cells, we performed single-cell RNA sequencing analyses of PBMCs from the 15 study subjects using the DNBSEQ-G400 platform (Figure 1A). After quality control and filtering, 120,876 cells were finally obtained, including 40,808 cells from the case group (5 subjects who became asymptomatic, chronic HBsAg carriers) and 80,068 cells from the control group (10 subjects who were negative for both HBsAg and anti-HBs) (Table 1). Twenty-two clusters were identified using an unsupervised clustering method (Figure 1B), annotated as 12 cell types, based on canonical marker gene expression, and their distributions were visualized using Uniform Manifold Approximation and Projection (UMAP) (Figure 1B,C), including B cells (CD79A+MS4A1+CD79B+), plasma cells (MZB1+JCHAIN+XBP1+), CD14+ monocytes (CD14+LYZ+FCN1+), CD16+ monocytes (AIF1+LST1+FCGR3A+), NK cells (FCGR3A+NCAM1+NKG7+GNLY+GZMH+), T cells (CD3D+CD3E+CD3G+), NKT cells (FCGR3A+NCAM1+NKG7+GNLY+GZMH+CD3D+CD3E+CD3G+), proliferative cells (TOP2A+MKI67+STMN1+), cDC (conventional dendritic cells) (CD1C+CLEC10A+FCER1A+), pDC (plasmacytoid dendritic cells) (IRF7+LILRA4+CLEC4C+), neutrophils (FCGR3B+CSF3R+G0S2+), and platelets (PPBP+PF4+GP9+) (Figure 1C,D). All 12 cell types could be seen in both the case and control groups. By further comparison, no significant difference was found between the two groups in the proportions of these 12 cell types. The *p* values are 0.16, 0.86, 0.68, 0.95, 0.59, 0.95, 0.68, 0.37, 0.31, 0.70,0.95 and 0.13 for B cells, plasma cells, CD14+ monocytes, CD16+ monocytes, NK cells, T cells, NKT cells, proliferative cells, cDC, pDC, neutrophils and platelets, respectively (Figure 1E).The proportion of immune cells varied among the samples; however, there was no significant difference in the proportions of immune cells (Figure 1F). 

### 3.3. Single-Cell Landscape of PBMCs, NKT Cells and T Cells

**Figure 2:**
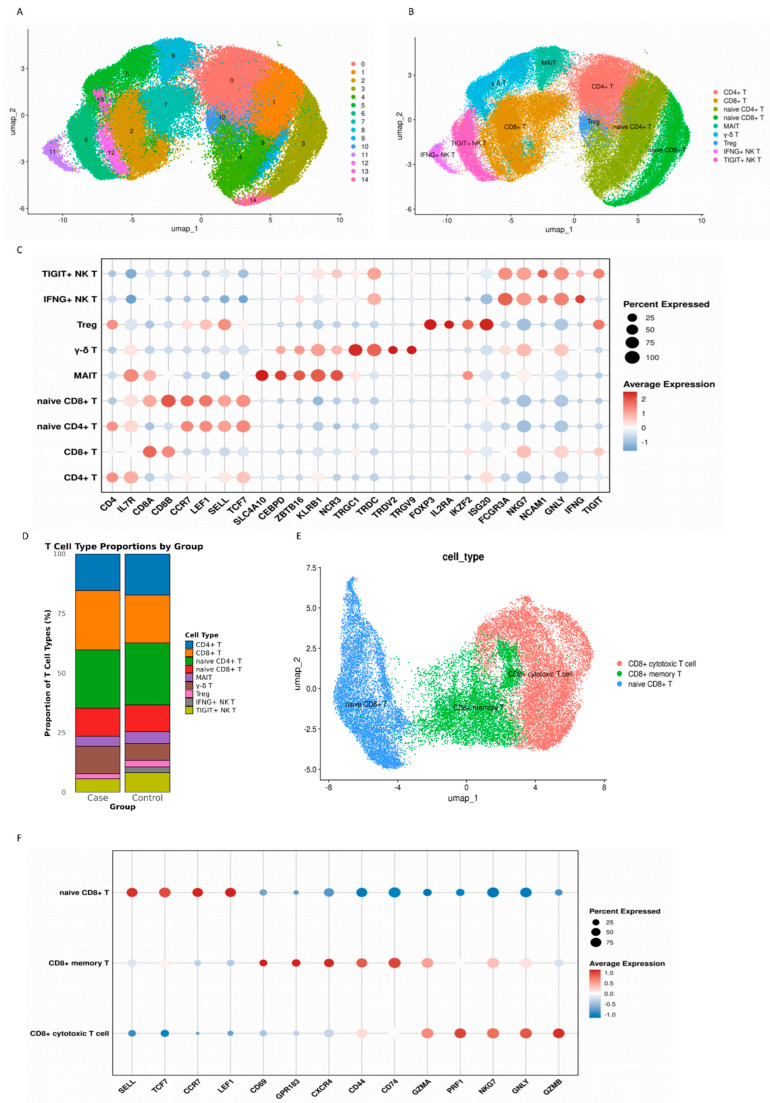
Single-cell transcriptome map of NKT cells and T cells: (**A**) UMAP plot of the 82,688 single cells from 15 individuals, revealing 15 distinct cell clusters. All 15 samples were merged and analyzed using Harmony for integration after normalization, variable feature selection, and scaling with the Seurat package. The clusters were identified through unsupervised clustering and visualized using UMAP; (**B**) UMAP plot visualizing the distribution of the 9 identified subpopulations of NKT and T cells in the integrated dataset from all subjects; (**C**) dot plots showing the expression of key marker genes (x-axis) across the identified NKT and T cell subpopulations (y-axis) in the integrated dataset from all subjects. The depth of the color from light to dark and the size of the dots represent the average expression from low to high and the percentage of cells expressing the gene; (**D**) bar chart showing the proportion of subpopulations of NKT and T cells in the case and control groups; (**E**) UMAP plot visualizing the distribution of the 3 identified CD8+ T cell subpopulations in the integrated dataset from all subjects; and (**F**) dot plots showing the expression of key marker genes (x-axis) across the identified CD8+ T cell subpopulations (y-axis) in the integrated dataset from all subjects. The notes are the same as in (**C**).

It has been reported that T follicular helper 17 (Tfh17) cells are critical for the maintenance of immunological memory [15]. Dimensionality Reduction Clustering was performed for NKT cells and T cells. Fifteen clusters were identified using an unsupervised clustering method and annotated as 9 cell types, based on canonical marker gene expression. Their distribution was visualized using UMAP (Figure 2A,B), including CD4+ T (CD4+IL7R+), naive CD4+ T (CD4+CCR7+LEF1+SELL+TCF7+), CD8+ T (CD8A+CD8B+), naive CD8+ T (CD8A+CD8B+CCR7+LEF1+SELL+TCF7+), MAIT (SLC4A10+CEBPD+ZBTB16+KLRB1+NCR3+), γ-δ T(TRGC1+TRDC+TRDV2+TRGV9+), Treg (CD4+FOXP3+IL2RA+IKZF2+ISG20+), TIGIT+ NK T (FCGR3A+NKG7+NCAM1+GNLY+TIGIT+) and IFNG+ NK T (FCGR3A+NKG7+NCAM1+GNLY+IFNG+) cells (Figure 2C). All 9 cell types were seen in both the case and control groups. There was also no significant difference in the proportions of these 9 cell types between the case and control groups (CD4+ T (*p* = 0.371); CD8+ T (*p* = 0.768); naive CD4+ T (*p* = 0.768); naive CD8+ T (*p* = 0.859); MAIT (*p* = 0.594); γ-δ T (*p* = 0.099); Treg (*p* = 0.44); IFNG+ NK T (*p* = 0.937); TIGIT+ NK T (*p* = 0.768)) (Figure 2D).

Dimensionality Reduction Clustering was performed for naive CD8+ T cells and CD8+ T cells. According to the expression of canonical marker genes, three cell types were identified by UMAP (Figure 2E,F), including naive CD8+ T (SELL+TCF7+CCR7+LEF1+), CD8+ memory T (CD69+GPR183+CXCR4+CD44+CD74+) and CD8+ cytotoxic T cells (GZMA+PRF1+NKG7+GNLY+GZMB+).

### 3.4. Single-Cell Landscape of B Cells

**Figure 3:**
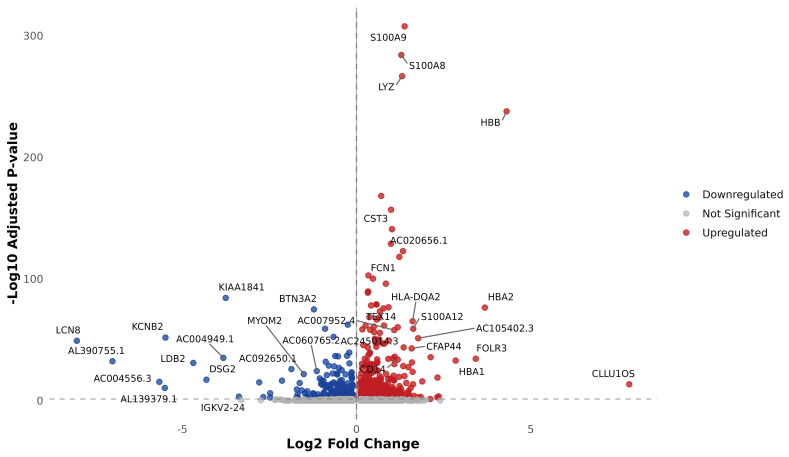
Volcano plot showing significantly upregulated and downregulated genes in B cells between cases and controls.

The total numbers of B cells captured in the case and control groups were 2366 and 6802, respectively. Dimensionality reduction clustering was performed for the B cells. According to the expression of canonical marker genes, four subpopulations of B cells were identified by UMAP: naive B cells, memory B cells, atypical memory B cells, and plasmablasts. The distributions of naive B cells, memory B cells, atypical memory B cells, and plasmablasts in the case group were 52.58%, 41.97%, 0.59% and 4.86%, respectively. The distributions of these cells in the control group were 47.94%, 46.28%, 0.01% and 5.76%, respectively. Complete differential expression analysis in B cells may be seen in Figure 3. The results of differential expression gene analysis of functional markers ((CD27, CD38, IGHG1 (IgG), IGHG2 (IgG), IGHG3 (IgG), IGHG4 (IgG), IGHM (IgM), IGHD (IgD), CXCR5, CD19, and MS4A1 (CD20)) showed that IGHG2 was significantly downregulated (avg_log2FC = −0.73, *p*.adj = 2.51 × 10^−14^).

### 3.5. Downregulated Expression of the IL7R and BACH2 Genes Was Associated with Immune Memory Loss

**Figure 4:**
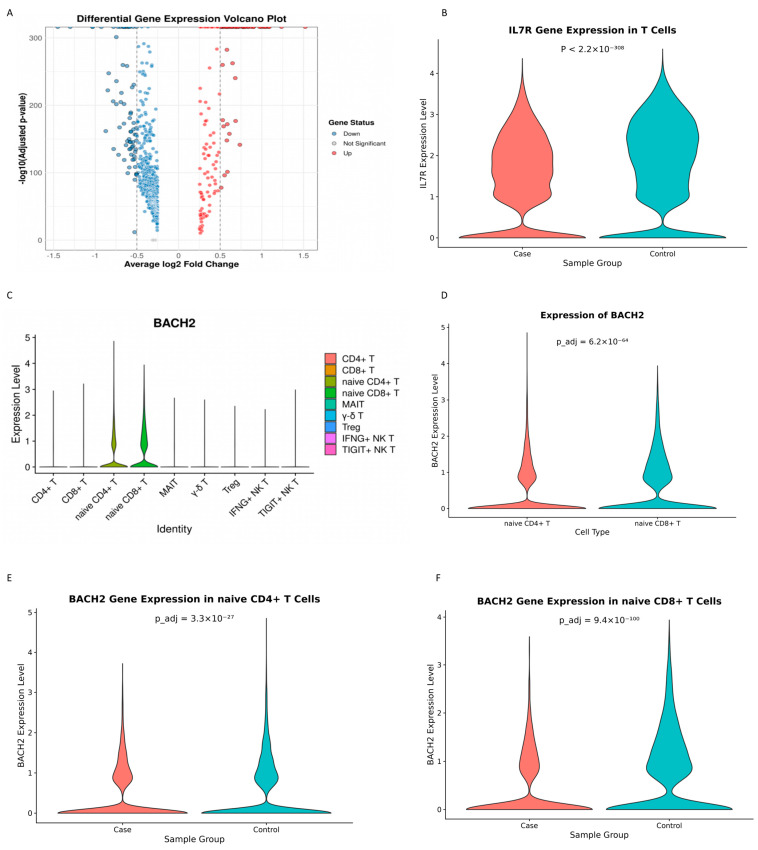

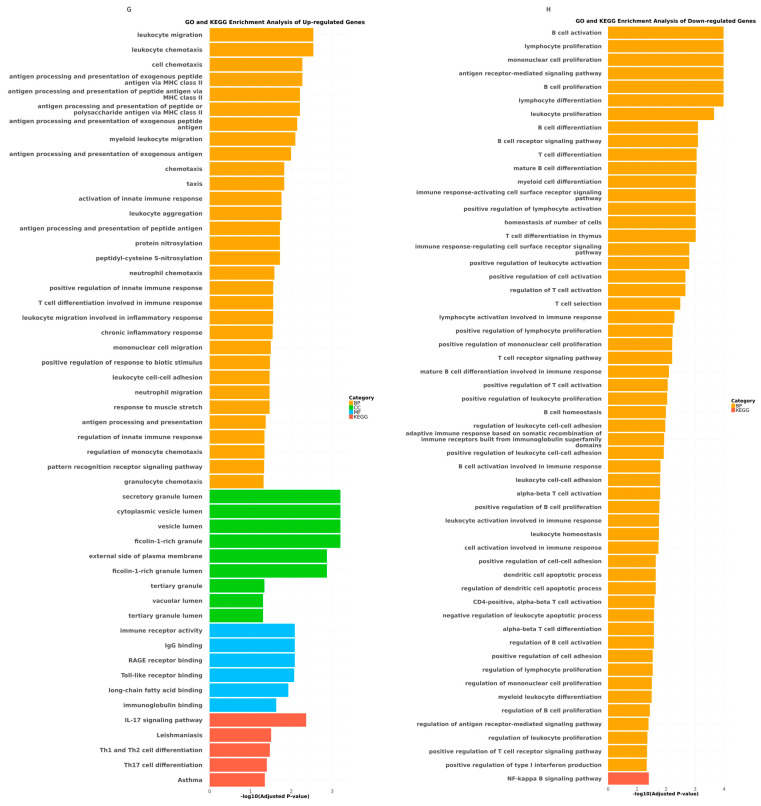
Differential gene expression (DGE) and functional enrichment: (**A**) volcano plot showing significantly upregulated and downregulated genes between cases and controls; (**B**) differential expression of the IL7R gene in T cells between cases and controls; (**C**) expression levels of the BACH2 gene across T cell subpopulations in the integrated dataset from all subjects; (**D**) differential expression of the BACH2 gene between naïve CD4+ and naïve CD8+ T cells in the integrated dataset from all subjects; (**E**) differential expression of the BACH2 gene in naïve CD4+ T cells between cases and controls; (**F**) differential expression of the BACH2 gene in naïve CD8+ T cells between cases and controls (Mann–Whitney U test); (**G**) GO and KEGG enrichment analysis of upregulated differential expression genes of T cells between the case and control groups; and (**H**) GO and KEGG enrichment analysis of downregulated differential expression of T cell genes between the case and control groups.

Differential gene expression analysis was performed for T cells, between the case and control groups, using the FindMarkers function in the Seurat package and identified 685 DEGs (|log2fc| > 0.25 and *P*adj < 0.05). Of the 685 genes, 150 were upregulated and 535 were downregulated in the case group, compared to the control group. A volcano plot for the DEGs is displayed in Figure 4A. 

Two of the DEGs, the interleukin-7 receptor (IL7R) gene and BTB domain and CNC homolog 2 (BACH2), have been reported to be associated with immune memory [16,17]. In this study, IL7R was found to be expressed in B cells, plasma cells, CD14+ monocytes, CD16+ monocytes, NK cells, T cells, NKT cells, proliferative cells, cDC, pDC, neutrophils, platelets and unassigned cells. Differential expression of IL7R gene could be seen in T cells, platelets, neutrophils, CD14+ monocytes and unassigned cells. However, only the expression levels in T cells (*p* < 2.2 × 10^−308^, including naïve CD4+ T, CD4+ T, naïve CD8+ T, CD8+ T, MAIT, γ-δ T and Treg) and CD14+ monocytes (*p* = 3.548 × 10^−7^) differed significantly between the case and control groups. Further analysis using the Mann–Whitney U test showed that the expression of IL7R in T cells was significantly downregulated in the case group, compared to the control group (*p* < 2.2 × 10^−308^) (Figure 4B), suggesting that downregulated expression of IL7R may be associated with immune memory loss.

BACH2 was found to be expressed in B cells, cDC, plasma cells, proliferative cells, T cells and unassigned cells. Differential expression of the BACH2 gene could be seen in T cells and unassigned cells. However, only the expression level in naive CD4+ T cells and naive CD8+ T cells differed significantly between the case and control groups (Figure 4C). Further analysis using the Mann–Whitney U test revealed that the expression level of the BACH2 gene in naive CD8+ T cells was significantly higher than that in naive CD4+ T cells (*p* = 6.21 × 10^−64^) (Figure 4D). The expression level of BACH2 in naive CD4+ T cells and naive CD8+ T cells was significantly lower in the case group than in the control group (*p* =3.31 × 10^−27^ and 9.41 × 10^−100^, respectively) (Figure 4E,F). Compared to the control group, BACH2 was downregulated 1.49-fold in naive CD4+ T cells in the case group, while it was downregulated 2.25-fold in naive CD8+ T cells in that group. These results suggest that downregulation of BACH2 in both naive CD4+ T cells and naive CD8+ T cells is associated with immune memory loss. The results of functional enrichment of DEGs using GO and KEGG pathway analysis showed that 46 DEGs were upregulated and 55 DEGs were downregulated in the GO analysis (covering biological processes, cellular components, and molecular functions), 5 DEGs were upregulated and 1 DEG was downregulated in the KEGG analysis (Figure 4G,H).

Considering that the subjects in the case group were persistently infected with HBV, in order to clarify whether the observed changes reflected alterations secondary to persistent infection, rather than the primary mechanisms of memory loss, we compared the expression levels of the IL7R and BACH2 genes of those subjects positive for anti-HBc between the case and control groups. The expression levels of both genes in the anti-HBc positive subjects were significantly lower in the case group (*p*.adj = 2.978368 × 10^−137^, 6.095209 × 10^−65^, respectively). Comparison of the expression levels of the two genes between subjects positive and negative for anti-HBc within the control group revealed that the expression of BACH2 was significantly lower in anti-HBc-positive subjects (*p*.adj = 1.89069 × 10^−9^) than in those negative for anti-HBc. These data suggest that downregulated expression of the IL7R and BACH2 genes is associated with immune memory loss and downregulation of BACH2 may also be a result of infection, because anti-HBc positivity indicates past or ongoing HBV infection.

### 3.6. Expression of the IL7R and BACH2 Genes at Various Stages of Cell Development

**Figure 5:**
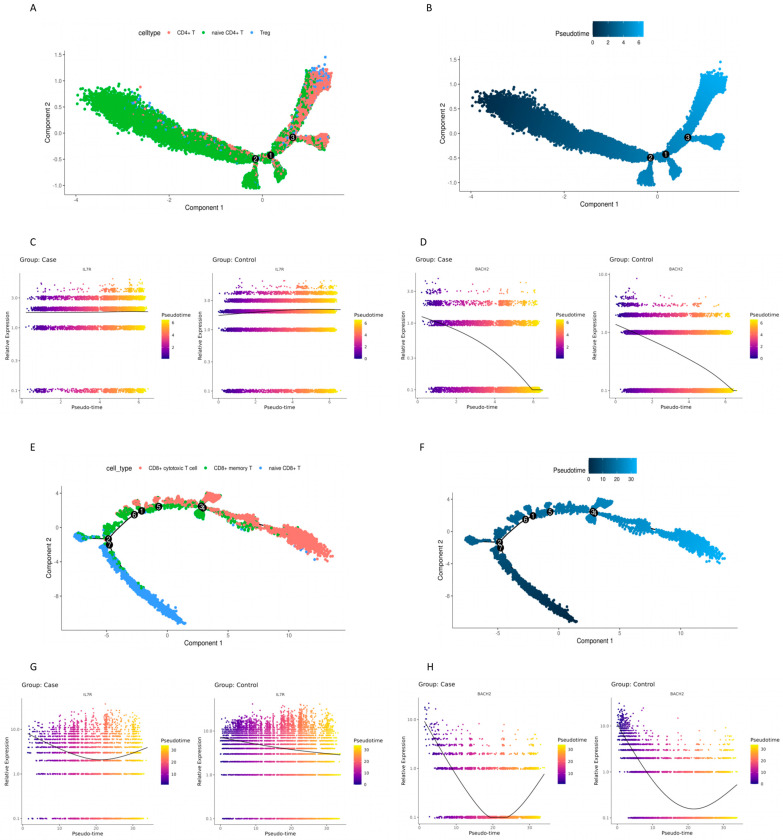
Pseudotime-ordered analysis of CD4+ and CD8+ T cells from cases and controls: (**A**) distribution of annotated CD4+ T cell subtypes along the pseudotime trajectory from the integrated dataset of all subjects; (**B**) pseudotime trajectory of the integrated CD4+ T cell population from the integrated dataset of all subjects; (**C**) IL7R expression dynamics of CD4+ T cells along the pseudotime in cases and controls; (**D**) BACH2 expression dynamics of CD4+ T cells along the pseudotime in cases and controls; (**E**) distribution of annotated CD8+ T cell subtypes along the pseudotime trajectory from the integrated dataset of all subjects; (**F**) pseudotime trajectory of the integrated CD8+ T cell population from the integrated dataset of all subjects; (**G**) IL7R expression dynamics of CD8+ T cells along the pseudotime in cases and controls. (**H**) BACH2 expression dynamics of CD8+ T cells along the pseudotime in cases and controls. Note: The numbers in black circles denote key nodes within the trajectory structure, representing bifurcation points of cell differentiation. These numbers function solely as node identifiers to distinguish different branch points and do not carry any fixed ordinal or hierarchical significance.

The Monocle tool (Version 2.34.0) was used to construct differentiation trajectories of the T cells. Trajectory analysis of CD4+ T cells inferred a differentiation direction that began with naïve CD4 T cell clusters, with a few CD4+ T cells and Treg cells, and split into three activation branches. Two branches maintained naïve CD4+ T cells as the major cell type while the other branch bifurcated into two sub-branches. Although the major cell type in the two sub-branches was CD4+ T cells, one sub-branch had more Treg cells (Figure 5A,B). The trend of IL7R gene expression is similar in the case and control groups, although the expression level in the controls was a little higher (Figure 5C). The trend of BACH2 gene expression is similar in the case and control groups (Figure 5D).

Trajectory analysis of CD8+ T cells inferred a differentiation direction that began with naïve CD8 T cell clusters and bifurcated into two activation branches. One branch contained naïve CD8+ T cells and CD8+ memory T cells. The other branch contained CD8+ memory T cells and CD8+ cytotoxic T cells. The latter branch split further into three activation sub-branches. One sub-branch contained CD8+ memory T cells and one contained CD8+ cytotoxic T cells. The third contained a mixture of CD8+ memory T cells and CD8+ cytotoxic T cells (Figure 5E,F). It could be seen in both the case and control groups that IL7R gene expression was high early in the pseudotime analysis, then decreased, and increased again only in the case group (Figure 5G), suggesting that T cells in the case group may not maintain a terminally differentiated state. BACH2 expression was highly early in the pseudotime analysis in both the case and control groups, then decreased and increased again at the end stage, suggesting that T cells in the case and control groups also may not maintain a terminally differentiated state (Figure 5H).

Clearly, the IL7R and BACH2 genes are expressed at different stages of cell development. IL7R is expressed throughout cellular development while BACH2 is expressed only at the early stage.

## 4. Discussion

To our knowledge, this is the first study to search for the factors associated with immune memory loss in the transcriptional profiles of immune memory cells from the peripheral blood of HepB vaccine recipients who become asymptomatic HBsAg carriers. The major findings are that downregulated expression of the IL7R and BACH2 genes in T cells is associated with immune memory loss. The expression levels of IL-7R in T cells and CD14+ monocytes and the expression of BACH2 in naive CD4+ T cells and naive CD8+ T cells were all significantly downregulated, compared to the control group. The expression of the IL7R gene occurred throughout the cellular development while that of the BACH2 gene only occurred in the early stage of cell development. A strength of this study is that the transcriptional profiles of immune memory cells were determined using scRNA-seq, so that transcriptional similarities and differences within a population of cells could be assessed. A major limitation is the small sample size, particularly within the case group, which was constrained by the cohort availability and cost. This limitation weakens the generalizability of the conclusions.

Cytokines IFN-γ and IL-5 are used frequently as markers of anamnestic memory; negativity for these cytokines indicates loss of immune memory [18]. It has been reported that 10–50% of immunized individuals did not achieve positive antibody levels after a one-dose booster, implying loss of immune memory [10,18,19]. However, only a few of such individuals become chronic HBV carriers [5,6]. What is the difference between those who become chronic HBV carriers and those who did not become persistently infected, among subjects negative for anti-HBs or with immune memory loss? This study is the first to address this issue.

When the HepB vaccine is administered, HBsAg is first taken up by antigen-presenting cells (e.g., dendritic cells [DCs]). DCs mature with the help of the local innate immune response and migrate into the lymph nodes. Mature DCs activate CD4+ and CD8+ T cells and some activated CD4+ helper T cells differentiate into follicular helper T (Tfh) cells. These induce B-cell differentiation into memory B cells and plasma cells (antibody-secreting cells) [20]. Long-term immunity is assured by memory B cells and T cells in the blood and lymph nodes, as well as by long-lived plasma cells and memory T cells in the bone marrow [11]. The cytokine IL-7 and its receptor, IL-7R, are a non-redundant growth, differentiation, and survival factor for human T lymphocytes. The differentiation and long-term survival of CD4 and CD8 memory T cells relies on IL-7/IL-7R [21]. IL-7R may be useful for predicting the number of memory T cells generated after infection or immunization [22]. Most circulating, mature T cells express IL-7R [23] and its expression may be a predictive marker of protection [22]. In this study, we found that the expression level of IL-7R in T cells was significantly lower in the case group than the control group. Here, some subjects in the control group were not currently infected with HBV, although they had become positive for anti-HBc, suggesting that they had not lost immune memory completely. These findings suggest a candidate gene for future functional studies to explore its potential role in the loss of immune memory and may inform adjuvant design if a causal mechanism is firmly established.

BACH2 is a pivotal transcription factor that is crucial for memory T cell differentiation during acute infection [24]. It promotes both the development and recall proliferation of memory CD8+ T cells through Prdm1 and Id3 [25]. It restrains terminal differentiation to enable the generation of long-lived memory cells and protective immunity following viral infection [17]. In this study, the expression level of BACH2 in T cells was found to be significantly lower in the case group than the control group. Clearly, downregulation of BACH2 is associated with immune memory loss. This finding also suggests a candidate gene for future functional studies, to explore its potential role in the loss of immune memory.

The activation of CD4+ T cells requires IL7R [26]. Some activated CD4+ Th cells differentiate into Tfh cells [20]. Tfh17 cells are critical for the maintenance of immunological memory [15]. In this study, although B cells, plasma cells, CD14+ monocytes, CD16+ monocytes, NK cells, T cells, NKT cells, proliferative cells, cDC, pDC, neutrophils, platelets and unassigned cells were found to express IL7R, only the expression levels in T cells differed significantly between cases and controls, suggesting that T cells are associated with immune memory loss.

It has been reported that, among T cell subpopulations, BACH2 is highly expressed in naive T cells, including naive CD4+ and CD8+ T cells, and downregulated upon effector/memory CD8+ T cell differentiation [27]. This was confirmed by our results. We found that BACH2 is highly expressed in naive CD4+ and CD8+ T cells but not in CD8+ memory T cells or CD8+ cytotoxic T cells. However, an apparent contradiction is that Bach2 maintains T cells in a naive state by suppressing effector memory-related gene, while downregulation of BACH2 is associated with immune memory loss.

BACH2 could have distinct, context-dependent functions in vaccine-induced immunity, compared to immunity induced by chronic infection [28]. It has been reported that BACH2 expression is upregulated in the early phases of memory T cell formation, which may contribute to establishing the immune memory induced by vaccines [29]. Downregulation of BACH2 in naive CD8 + T cells is essential for the complete differentiation of effector cells [30]. Therefore, the plausible mechanism of the association of downregulation of BACH2 with immune memory loss may be that HepB vaccine cannot induce long-term immune memory because of its downregulation in naive T cells. However, the possibility that downregulation of BACH2 may be a result of infection could not be excluded. Further study is required to confirm these possibilities.

The concurrent downregulation of IL-7R and BACH2 is a plausible phenomenon. It has been reported that BACH2 ablation decreases the expression of IL-7R in long-lived memory cells, reduces the proportion of central memory T (Tcm) cells, and increases that of effector memory T (Tem) cells in gut primary CD4+ and CD8+ T populations, compared with nontargeting controls. This suggests that BACH2 acts upstream of IL-7R [31]. Strong or prolonged TCR signaling, inflammatory cytokines, or exhaustion cues repress BACH2 to enable effector programs (e.g., by granting AP-1 factors access to enhancers). This loss of BACH2 subsequently drives secondary IL-7R downregulation, which reduces sensitivity to IL-7-mediated survival and proliferation signals and creates a feed-forward loop that favors terminal differentiation over memory- or stem-like fates [32]. Therefore, we hypothesize that dysregulation of a common upstream signaling pathway, such as STAT5, might lead to the coordinated downregulation of both IL7R and BACH2, thereby impairing memory T cell survival and transcriptional programming. However, this remains to be confirmed.

No gene related to immunological memory loss could be found in memory B cells, plasmablasts, although attempts were made to identify differentially expressed genes (DEG) with different *p* values. Might the cause of immunological memory loss be attributed to T cells rather than B cells or plasmablasts? It is notable that only circulating B cells were analyzed, and not those in lymph nodes and long-lived plasma cells. These require further study. Although this study has produced some interesting findings, validations with larger cohorts, and especially in other regions, are required.

## 5. Conclusions

Our results highlight the association of expression levels of the IL7R and BACH2 genes in T cells and their subpopulations with immune memory loss to the HepB vaccine. These results suggest candidate genes for future functional studies to explore their potential role in the loss of immune memory and could inform adjuvant design if a causal mechanism is firmly established.

## Figures and Tables

**Table 1:** Baseline information of the study subjects.

Code	Groups	Gender	Ages	HBsAg/Anti-HBs (2009) §	HBsAg/Anti-HBs (2015)	Anti-HBc (2015)	HBsAg/ Anti-HBs (2017)	Anti-HBc (2017)	HBsAg/ Anti-HBs (2024)	HBeAg/ Anti-HBe (2024)	Anti-HBc (2024)	Levels of HBsAg in 2024 (mIU/mL)	Number of Cells Captured and Sequenced
ADB201	Case	M *	34	+/−	+/−	+	+/−	+	+/−	−/−	+	250	8344
ANL008	Case	M	37	ND ^&^	+/−	+	+/−	+	+/−	−/−	+	250	8017
ATW052	Case	F ^#^	34	+/−	−/+	−	+/−	+	+/−	−/+	+	250	7509
ATY094	Case	M	32	ND	−/+	−	+/−	+	+/−	−/+	+	ND	7352
AYH125	Case	F	35	ND	+/−	+	+/−	+	+/−	−/−	+	134	9586
ACS025	Control △	F	34	ND	ND ^&^/ND	ND	−/−	−	−/−	−/−	−	ND	6253
ANT046	Control	F	36	−/+	ND/ND	ND	−/−	−	−/−	−/−	−	ND	9128
ANF175	Control	F	36	−/+	−/+	+	−/−	−	−/−	−/−	−	ND	5656
ANF082	Control	M	37	−/−	−/−	−	ND/ND	ND	−/−	−/−	−	ND	8844
09LA1142	Control	M	37	−/−	ND/ND	ND	−/−	−	−/−	−/+	−	ND	6753
ANL070	Control	M	36	ND	ND/ND	ND	−/−	−	−/−	−/−	+	ND	8823
ANS124	Control	M	35	−/+	ND/ND	ND	−/−	−	−/−	−/−	−	ND	9778
ANS385	Control	M	31	ND	−/−	+	ND/ND	ND	−/−	−/−	−	ND	6334
ANS040	Control	M	36	−/+	ND/ND	ND	−/−	+	−/−	−/−	−	ND	8612
09LA1232	Control	M	34	−/+	−/−	−	ND/ND	ND	−/−	−/−	−	ND	9887

§: Dates in parentheses indicate the year of testing. M *: Male, F ^#^: Female, ND ^&^: Not done. △: For the other five control subjects, previous positivity for anti-HBs was documented in local hospital records.

## Data Availability

The original contributions presented in this study are included in the article. Further inquiries can be directed to the corresponding author.
